# Organoids derived from patients provide a new opportunity for research and individualized treatment of malignant peritoneal mesothelioma

**DOI:** 10.1186/s12943-023-01901-z

**Published:** 2024-01-10

**Authors:** XiaoChang Fang, Lin Shu, TianLiang Chen, XiaoLe Zhao, LiuCui Yang, Tingting Dou, Lijie Yang, Xuanfei Li, Maohui Feng

**Affiliations:** 1 Department of Gastrointestinal Surgery, Zhongnan Hospital of Wuhan University, Wuhan, China; 2 Clinical Medical Research Center of Peritoneal Cancer of Wuhan, Wuhan, China; 3 Clinical Cancer Study Center of Hubei Provence, Key Laboratory of Tumor Biological Behavior of Hubei Provence, Wuhan, China; 4 Union Hospital, Tongji Medical College, Huazhong University of Science and Technology, Wuhan, China

**Keywords:** Malignant peritoneal Mesothelioma, Organoids, Patient-derived organoids (PDO), Primary cell lines, Patient-derived organoids xenograft (PDOX), Peritoneal orthotopic xenograft, Precision medicine, Translational medicine, Drug screen

## Abstract

**Background:**

Malignant peritoneal mesothelioma (MPM) is an extremely rare and highly invasive tumor. Due to the lack of accurate models that reflect the biological characteristics of primary tumors, studying MPM remains challenging and is associated with an exceedingly unfavorable prognosis. This study was aimed to establish a new potential preclinical model for MPM using patient-derived MPM organoids (MPMOs) and to comprehensively evaluate the practicality of this model in medical research and its feasibility in guiding individualized patient treatment.

**Methods:**

MPMOs were constructed using tumor tissue from MPM patients. Histopathological analysis and whole genome sequencing (WGS) were employed to determine the ability of MPMOs to replicate the original tumor's genetic and histological characteristics. The subcutaneous and orthotopic xenograft models were employed to assess the feasibility of establishing an in vivo model of MPM. MPMOs were also used to conduct drug screening and compare the results with retrospective analysis of patients after treatment, in order to evaluate the potential of MPMOs in predicting the effectiveness of drugs in MPM patients.

**Results:**

We successfully established a culture method for human MPM organoids using tumor tissue from MPM patients and provided a comprehensive description of the necessary medium components for MPMOs. Pathological examination and WGS revealed that MPMOs accurately represented the histological characteristics and genomic heterogeneity of the original tumors. In terms of application, the success rate of creating subcutaneous and orthotopic xenograft models using MPMOs was 88% and 100% respectively. Drug sensitivity assays demonstrated that MPMOs have different medication responses, and these differences were compatible with the real situation of the patients.

**Conclusion:**

This study presents a method for generating human MPM organoids, which can serve as a valuable research tool and contribute to the advancement of MPM research. Additionally, these organoids can be utilized as a means to evaluate the effectiveness of drug treatments for MPM patients, offering a model for personalized treatment approaches.

**Supplementary Information:**

The online version contains supplementary material available at 10.1186/s12943-023-01901-z.

## Introduction

Malignant peritoneal mesothelioma (MPM) is a highly malignant and exceedingly rare tumor. This disease is classified by the World Health Organization into epithelioid, sarcomatoid, and biphasic types [1, 2]. Unlike pleural mesothelioma, peritoneal mesothelioma are less associated with asbestos exposure and often exhibit epithelioid tissue types [3–7]. Due to the rarity of peritoneal mesothelioma, which is characterized by diverse symptoms and a lack of specific characteristics, posing a significant barrier to the early diagnosis of MPM [8–13]. Consequently, the majority of patients are in the terminal phase at the time of diagnosis, leading to a median survival of only 5–12 months [14, 15]. Despite the recommendations of the Peritoneal Surface Oncology Group International (PSOgi), the combination of cytoreductive surgery (CRS) and hyperthermic intraperitoneal chemotherapy (hiPeC) as the primary treatment strategy can increase the median survival period of patients to 31–92 months [16–21]. However, there are still patients who have lost the opportunity for CRS treatment due to a late diagnosis, resulting in a poor prognosis. Currently, there is no effective treatment for these patients [20, 21].

In the past, a number of risk factors for the occurrence and progression of MPM have been proposed in research [22–29]. However, many issues, such as major pathogenic factors, key driving pathways, and drug genetic targets, particularly in various histological subtypes, remain unresolved. This may be due to the rarity of this illness and the absence of a research model for MPM. Malignant pleural mesothelioma cell lines and xenotransplantation models are the most frequently used instruments to study MPM at present, but their application is limited by drawbacks such as a long induction period, a low induction rate, and their unsuitability for drug screening [30]. In addition, malignant pleural mesothelioma and MPM may have distinct profiles of gene expression and genetic backgrounds [31, 32]. Therefore, it is essential to develop a preclinical model of MPM in order to further investigate the disease’s mechanism, identify new therapeutic targets, and conduct drug screening.

Organoids are a form of micro-organ that utilizes in vitro three-dimensional culture technology to cultivate specific stem cells that closely resemble the source tissue or organ [33]. Patient-derived organoid compounds (PDO) have been successfully applied to the study of multiple tumors, including breast [34], colorectal [35], stomach [36], gastrointestinal [37], prostate [38], lung [39], liver [40], pancreas [41], and nasopharyngeal [42]. Numerous studies have demonstrated that the pathological phenotype and genotype of PDO are consistent with the primary tumor tissue and that the prediction of drug efficacy closely resembles the drug response of clinical patients [34–42]. Therefore, it has been demonstrated that PDO can reproduce the actual situation of patients in vitro and more precisely predict the effect of treatment on patients. However, MPM-related organoid research has not yet been conducted.

In this study, we reported the generation and detailed analysis of MPM organoids from patients and showed that their morphology, histopathology, and genome landscape were consistent with the original tumor. In addition, we investigated the usefulness of these MPM organoids for investigating drug reactions. The results demonstrate that the newly established MPM organoids have the potential to predict the response of each patient to treatment and serve as a strategy for future MPM research or clinical applications.

## Materials and methods

### Human samples

From January 2020 to December 2022, tissue was obtained from patients with malignant peritoneal mesothelioma (MPM) who underwent surgery or a biopsy at the Zhongnan Hospital of Wuhan University. The histopathology of the resected MPM specimens was routinely evaluated by the Pathology Department of Zhongnan Hospital of Wuhan University and confirmed by two hospital pathologists. In the end, only the histopathology-confirmed MPM specimens were included in this study. The collected MPM samples are placed in a cold organoid preservation solution and conveyed on ice before being processed in accordance with the MPM organoid culture method. This research was approved by the Medical Ethics Committee of Zhongnan Hospital of Wuhan University (China) (No. 2015022, 2021054 and 2023005) and was conducted according to the Helsinki Declaration. Prior to the commencement of the study, all patients signed a written informed consent form. For each patient detailed information is shown in Supplementary Table 1.

### Organoid culture

MPM samples were washed three times with cold phosphate buffered saline (PBS) and minced into three small pieces with. A small random piece was fixed in 10% neutral-buffered formalin for histopathological analysis and immunofluorescence (IF) staining. One piece of minced tissue were snap frozen and stored at − 80 °C for DNA isolation, while the rest was used for the isolation of cancer cells. The MPM tissues for the derivation of organoids were further minced into < 3 mm pieces. Then, dynamically clean it with PBS containing 5% penicillin/streptomycin (15070-063, Gibco, USA) and 2% primocin (ant-pm-05, InvivoGen, France) for 5 min, 2.5% penicillin/streptomycin and 1% primocin for 5 min, and then rinse it 3 times with PBS containing 1% penicillin/streptomycin and 1% primocin. Collagenase Type IV (1 mg/mL, CAT # 7909, Stemcell, Canada) and hydroxyuronidase (0.2 mg/mL, HY107910, MedChemExpress, USA) were used to dissociated MPM tissues for 60–90 min at 37 °C. During digestion, vigorously agitate every 10–15 min. When no obvious tissue is observed or a mixture of cell clusters is observed under the microscope, add 10 times the volume of PBS for dilution, and then execute differential centrifugation (200×g, 5 min) ,remove the supernatant. Add cold advance DMEM/F12 resuspended sediment, filter the suspension with a 70 µm cell filter to remove larger tissues, and then remove the supernatant via differential centrifugation (200×g, 5 min). Sedimentation of 5 × 10^5^ single cells/cell clusters suspended in 40µL Cultrex Reduced Growth Factor Basement Membrane Extract, Type 2 (BME, 3536-005-02, R&D system, USA) and inoculated into preheated 48-well plates. Invert the inoculated 48-well plate and incubate it for 30 min at 37 °C. Wait until BME has completely solidified and formed a dome, then add 300 µL of MPMO’s culture medium and resume cultivation in an incubator at 37 °C and 5% CO_2_. The detailed information of MPMOs culture media is listed in Supplementary Table 2. The culture medium of MPMOs should be changed and photographed every two days.

When MPMOs reach 100–150µm, passage is possible. Remove the passable MPMOs culture medium and add 500 µL Cultrex Organoid Harvesting Solution (3700-100-01, R&D system, USA) was incubated at 4 ° C for 30 min to remove the BME surrounding the MPMOs, then 5mL AdDMEM/F12 was added for dilution, and the separated MPMOs were centrifuged at 200 g for 5 min to remove the supernatant. Suspend the isolated MPMO in BME and inoculate it at a ratio of 1:2 or 1:3, approximately every 14–20 days. To freeze-preserve MPMOs, they were dissociated from BME into minute clusters before being frozen in cell-free medium (C2874, Sigma Aldrich, USA).

### Primary cell culture

The MPM single cells/cell clusters that had been digested and filtered were collected and resuspended in Roswell Park Memorial Institute 1640 (RPMI1640) medium containing 15% fetal bovine serum (FBS), 1% penicillin/streptomycin, and 1% primocin. The cells were inoculated into a T25 flask and cultivated at 37 °C and 5% CO_2_. When the cells reach 80% growth, they are passage in a 1:2 ratio.

### Patient-derived organoids xenografts

For subcutaneous and abdominal xenotransplantation, four to six-week-old BALB/c nude mice (Sibeifu Beijing, China) devoid of specific pathogens were selected. The transplanted mice were housed in a non-pathogenic environment with a temperature range of 20–26 °C and a relative humidity range of 40-70%. Collect 5 organoid pores and resuspension them at 100 µL MPMOs medium. Select the armpit of Nude mice as the puncture point for subcutaneous xenotransplantation. After injection of MPMOs, measure subcutaneous tumors with a caliper every three days and calculate the tumor volume using the following formula: V = (Width^2^ ×Length) ÷ 2. After 15 days, remove the tumor and record its weight. Before injecting MPMOs into Nude mice for intraperitoneal orthotopic xenotransplantation, the mice were weighed and recorded. The lower left abdomen was designated as the puncture site. Nude mice injected with MPMOs were weighed every three days. After 15 days, transplanted Nude mice were executed, and tumor growth was confirmed via autopsy and experimental peritoneal cancer index (ePCI) [43] was performed.

### Histology and immunofluorescence

MPM tissue and subcutaneous xenograft tissue were fixed in 10% neutral buffered formalin for 24 h, dehydrated, embedded in paraffin, and serial sectioned at 6µm in thickness. Collect up to 100–150 µm size MPMOs and preserve them in 4% polyformaldehyde (PFA) for 1 h. The MPMOs were centrifuged for 5 min at 200 g to remove the supernatant. 3% agarose gel was added for resuspension, followed by paraffin embedding and serial sectioned at 6 µm - 8 µm in thickness. For Hematoxylin and eosin (H&E) staining, paraffin sections were dewaxed with xylene and rehydrated with ethanol gradients. For IF staining, the sections were dewaxed, rehydrated, and incubated in boiling EDTA solution (pH 8.0) for antigen restoration prior. Then, block for 2 h with 5% BSA and incubate overnight at 4 ° C with diluted primary antibodies (Supplementary Table 3). After incubation, wash the slide three times with PBST and incubate it at room temperature for two hours with diluted second antibodies (Supplementary Table 3), the nucleus was re-stained with DAPI. For IF staining of 2D cells, the cells were seeded onto a confocal culture dish 24 h before. Then, the culture medium was removed, washed twice with PBS, and fixed for 30 min with 4% PFA. After fixation, the plates should be cleaned three times with PBST, blocked for 1 h with 5% BSA. Following the completion of the blockage, primary antibody, secondary antibody, and DAPI staining are performed.

### DNA-sequencing analysis

For whole genome sequencing analysis, DNA was extracted from parental tumor tissues and related organoids using the DNeasy Blood and Tissue Kit (Qiagen) according to instructions by the manufacturer. For the preparation of DNA library, simply put, after the DNA quality control is qualified, it is subjected to ultrasonic DNA splicing, end repair, addition of “A”, and addition of DNA sequencer specific connectors. The DNA library is assembled via PCR enrichment and quantified using Qubit fluorescence quantification/enzyme-linked immunosorbent assay. Ultimately, double-stranded DNA was denatured, cyclized, and digested to produce single-stranded circular DNA, and DNA Nano Ball (DNB) were obtained by Rolling Circle Amplification (RCA). After the DNA library has been constructed, quantitative quality control is performed using Qubit before sequencing is conducted on the machine. Raw data obtained from sequencing is converted to sequenced reads via base calling analysis, then filtered and quality controlled to obtain clean reads. Use software BWA [44] to compare clean reads with the GRCh37 reference genome, followed by sorting [45] and labeling repeat sequences [46] in order to obtain the final alignment result. Copy Number Variant (CNV) analysis and extraction of potential polymorphic Single Nucleotide Polymorphism (SNP) sites were conducted using the mutation detection software Genome Analysis Toolkit (GATK) (version 4.1) [46], followed by filtering and screening using the bcftool2 program. The VEP software was used to predict and annotate mutation effects [47].

### Drug screen

For drug sensitivity testing, MPMOs were extracted 7 days after passage. The organoids also were resuspended in 2% BME/organoid complete medium (2 × 10^5^ organoids/mL) and plated in three equal portions in 96-well plates. The MPM primary cells were collected and resuspended in RPMI 1640 medium (2 × 10^5^ cells/mL), and inoculated in 96 well plate. Each chemotherapeutic drug was tested at eight different concentrations (0, 0.1, 0.5, 1, 5, 10, 50, and 100 M) for drug screening.

All of these medications are utilized in first- or possible second-line MPM treatment regimens (Supplementary Table 4) [19, 48–50]. After 48 h of incubation, cell viability was determined using CCK8 according to the manufacturer’s instructions, and absorbance at 450 nm was measured using a microplate reader. The half-maximal inhibitory concentration (IC50) values were calculated by nonlinear regression analysis of the dose–response curve.

### Statistical analysis

At least two independent trials of each experiment were conducted, with summary data presented as the mean standard deviation (SD). Sample size (n) are provided in the figures. For statistical analysis and image processing, the following software was utilized: GraphPad Prism 9.0.0 (GraphPad-Software, USA), Adobe Photoshop 2021 (Adobe Systems, USA), and Adobe Illustrator 2020 (Adobe Systems, USA). The IC50 were calculated through nonlinear regression. Student’s t-test was used for statistical significance evaluation. P < 0.05 indicates statistical significance.

## Results

### Generation of MPMOs and primary cell from patient samples

**Fig. 1:**
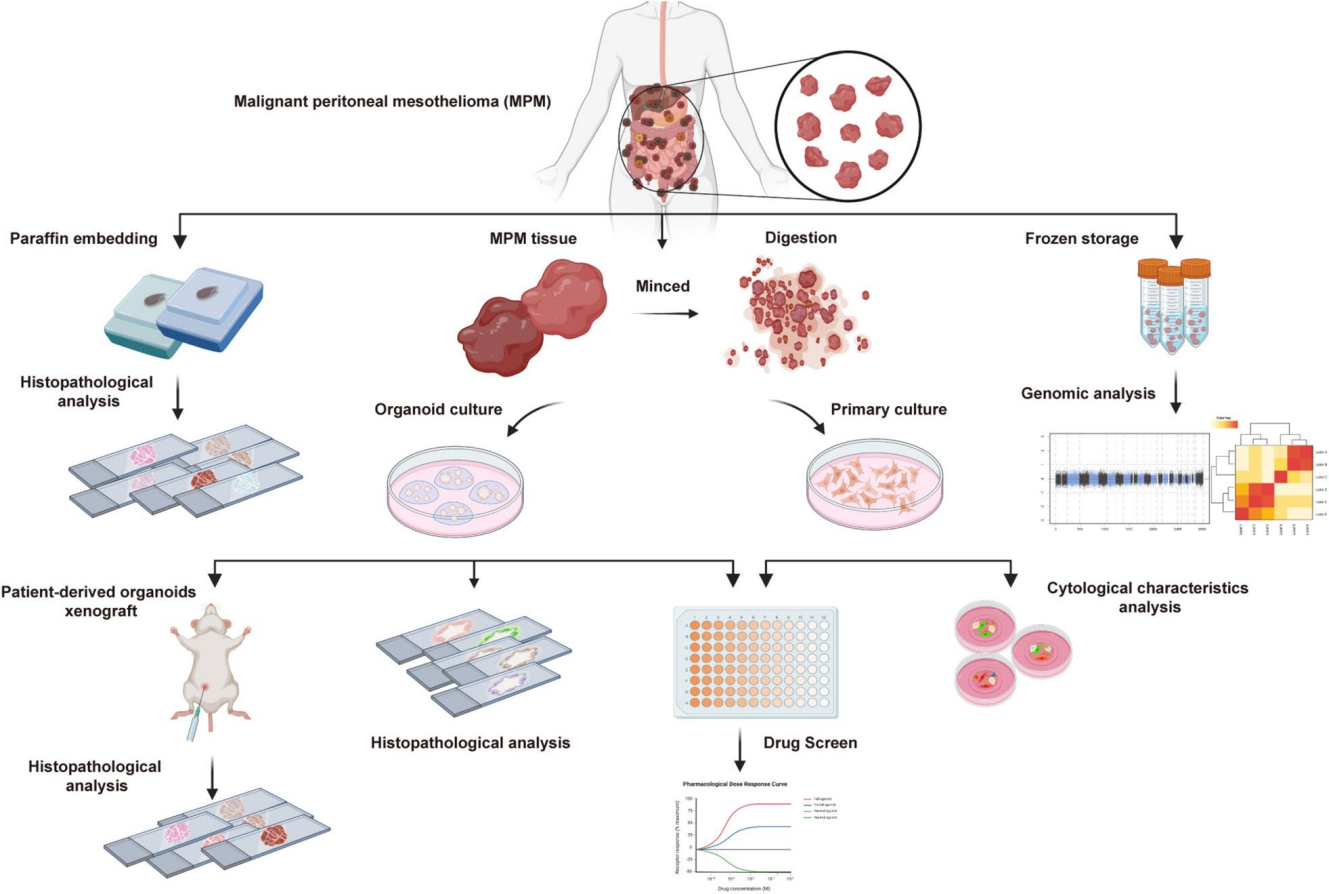
Flow diagram of establishment and characterization of MPMOs. Including the generation of MPMOs and primary cell from patients, as well as the histological characterization, genomic analysis, drug screen, and xenografts models of MPMOs.

**Fig. 2:**
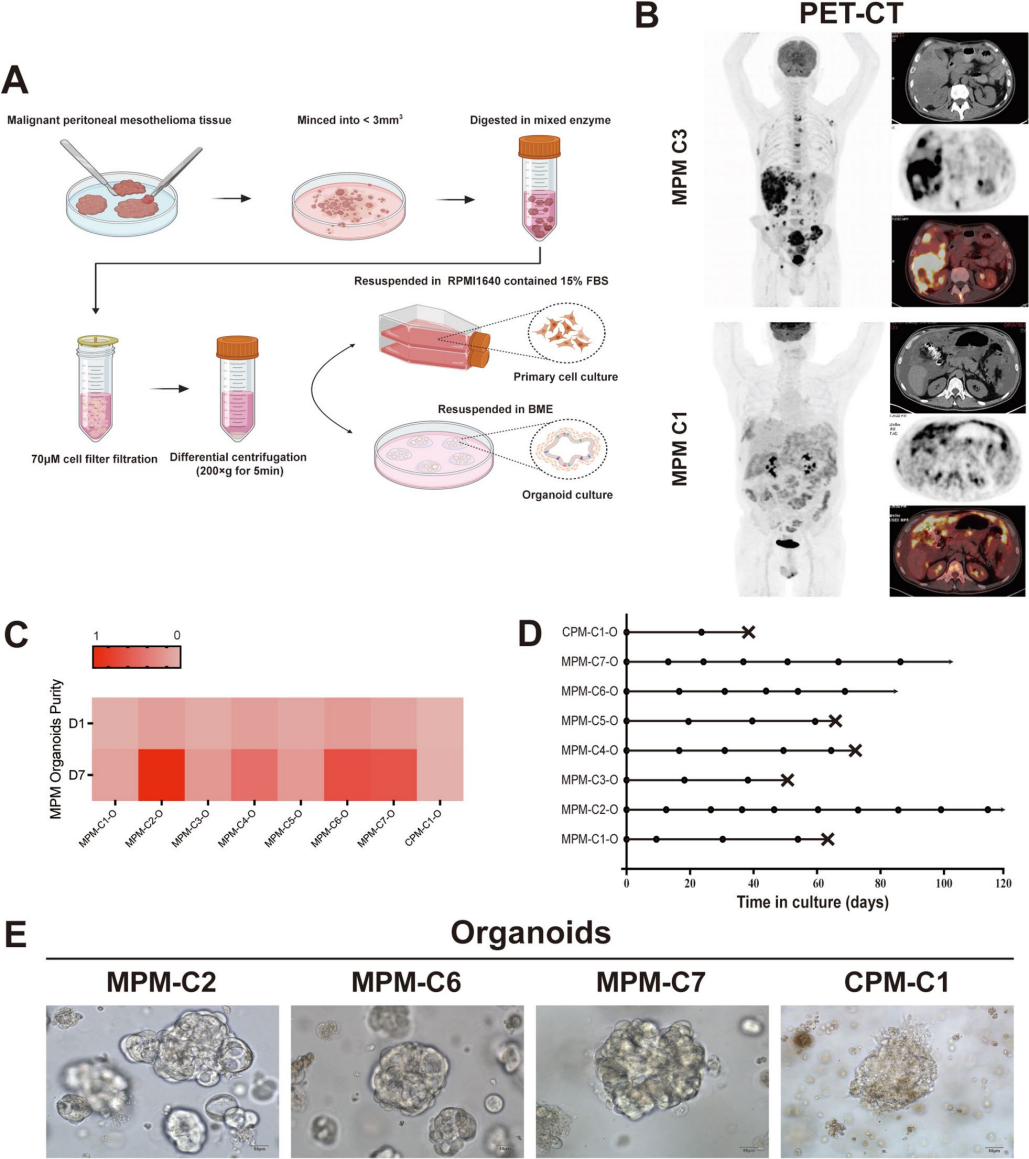
Establishment of patient-derived Malignant peritoneal mesothelioma (MPM) organoid cultures. **A** Representative images of MPMOs culture process, including the generation of MPM primary cells. **B** The PET/CT images of MPM patients showed significantly enhanced dense shadows in multiple areas of the abdominal cavity, and there were radioactive concentrations of imaging agents, indicating that the patient’s abdominal cavity has been invaded by multiple tumors. **C** Heat map showing the fraction of the derived organoids in the D1 and D7. **D** Expansion potential of MPM- and CPM-derived organoid. Arrow, continuous expansion ; Dot, passage ; Cross, Stop growth/death; MPM, malignant peritoneal mesothelioma ; CPM, cystic peritoneal mesothelioma ; O, organoid. **E** Representative bright field images of MPM and CPM-derived organoids from 4 patients. Scale, 50 μm

Malignant peritoneal mesothelioma (MPM) tissues were collected and divided for malignant peritoneal mesothelioma organoid (MPMO) culture, MPM primary cell culture, histopathology analysis, and genomic analysis. The drug sensitivity assays utilized MPMOs and MPM primary cells that were successfully cultured. Additionally, MPMOs were utilized to develop PDOX as a model for MPM in vivo research (Fig. 1). Each MPM tissue was minced into small pieces and enzymatic digestion was used to isolate cells/cell clusters. Post digestion, the cell/cell clusters were filtered through 70 µm cell strainers. For the culture of MPMOs, the cells/cell clusters were resuspended in BME and covered with organoid culture medium. For primary cell culture, resuspend cells/cell clusters in RPMI1640 medium containing 15% FBS and plant in T25 (Fig. 2A).

In our investigation, we obtained 8 samples from patients who underwent cell reduction surgery or biopsy, including 7 patients with MPM and 1 patient with cystic peritoneal mesothelioma (CPM) (Supplementary Table 1). Clinical PET/CT images of the patients show tumors and fuses in the omentum, mesentery, splenic capsule, liver, and pelvic peritoneum. Depending on the patient, abdominal tumors, ascites, and abdominal organs such as the liver and intestines may manifest to different degrees. Some patients may present with distant metastases, including lung, bone, and lymph nodes (Fig. 2B).

We established organoids and primary cells from seven MPM patients and one CPM patient. On the first and seventh days of cultivation, we counted the number organoids and compared their growth rates. On the seventh day of cultivation, the density of constructed organoids had already doubled, with MPM-C2-O exhibiting the most rapid growth (Fig. 2C). In addition, we recorded culture time and passage numbers of organoids (Fig. 2D), in which MPM-C2-O, MPM-C4-O, MP-C6-O, and MPM-C7-O were successfully cryopreserved and retested after more than four passages. Unfortunately, the CPM-O culture that we established in this study initially appeared to be successful, but it ceased growing after drug sensitivity and passage, the normal peritoneal organoids which culture from normal peritoneal tissues have not established yet.

From a morphological standpoint, the average diameter of MPMOs ranges between 120 and 250 μm. Multiple cystic structures resembling grapes are most prevalent in MPMOs, while spore-like structures can be observed in a few MPMOs. For CPM organoid, the morphology consists primarily of dense solids with numerous small cavities, similar to insect erosion structures (Fig. 2E).

#### Composition and culture characteristics of MPMOs culture medium

**Fig. 3:**
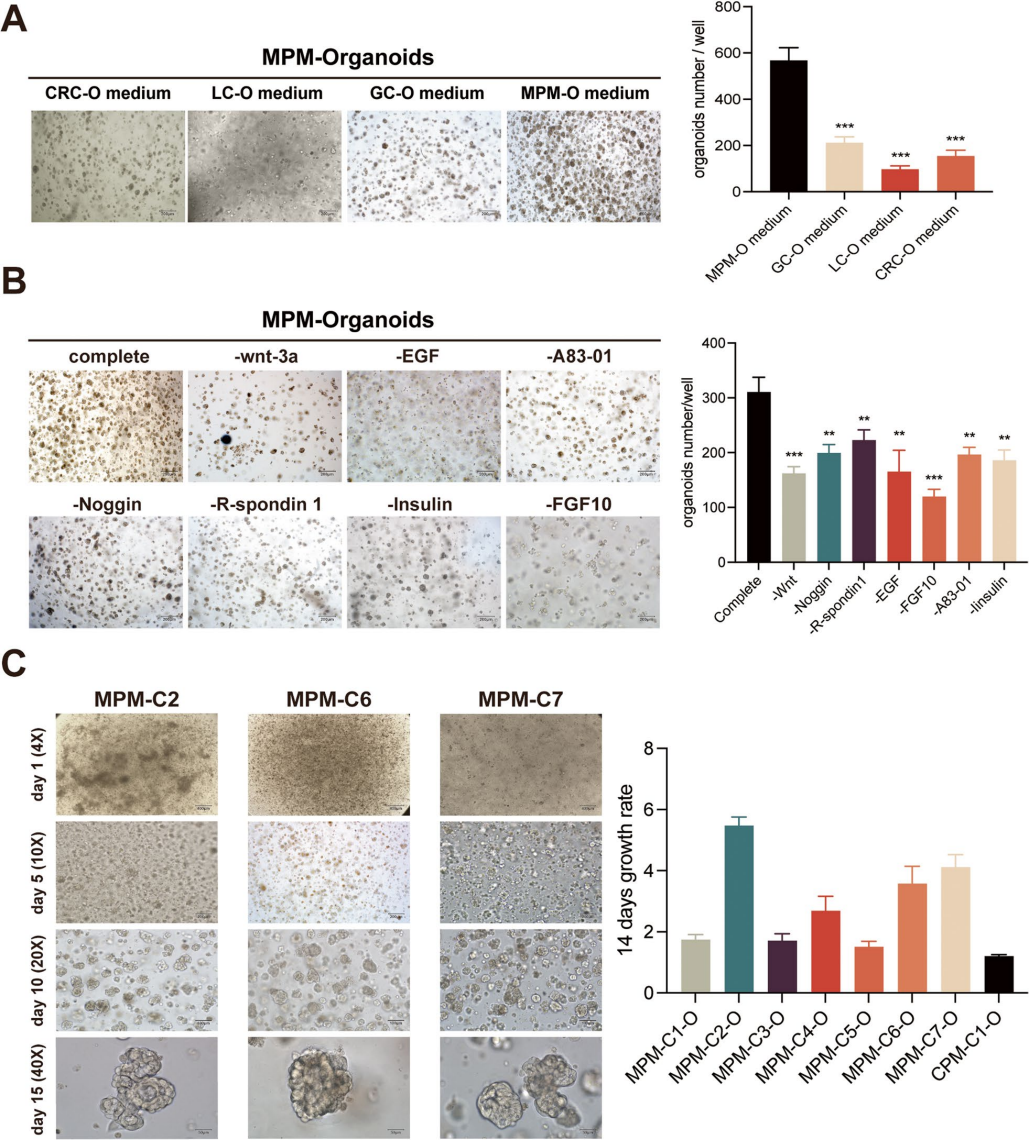
Culture characteristics of MPMOS and composition of culture medium. (A) MPMOs formed in different tumor-organoid culture medium. The image shown is from MPM-C1-O as a representative sample after 10 days of planting. Left, bright field images, scale, 200 μm;Right, Quantification of the MPMOs number per well in the different tumor-organoid culture medium. (B) The formation of MPM organoids culture in factor deletion medium after 10 days. The images shown are from MPM-C2-O as a representative sample. Left, bright field images, scale, 200 μm;Right, Quantification of the MPMOs number per well. (C) Time-lapse photography of images with different magnification of MPMOs. Left, bright field images, Scale bar, 400 μm, 200 μm, 100 μm, 50 μm;Right, Quantification of the MPMOs number per well for individual organoid. Data were showed as mean ± SD, *P < 0.05, **P < 0.01, ***P < 0.001 and each experiments were performed in triplicate

Due to the dearth of published research on MPMO, we are currently evaluating mature organoid culture media to determine the requirements for establishing MPMO. First, MPMOs were cultivated using previously reported media for human colorectal cancer (CRC) [35], gastric cancer (GC) [36], and lung cancer (LC) [39] organoids. Although MPMOs exhibited varying degrees of growth in different culture media, their growth was most pronounced in GC and CRC organoids culture media, as observed under a microscope. The number and size of MPMOs in these two culture media differed significantly from those in LC organoids culture media. We therefore tried to combine GC and CRC organoids culture media for the cultivation of MPMOs. As predicted, the combination of GC and CRC organoids culture media substantially increased the growth of MPMOs, and the growth capacity of MPMOs was greater than when only one culture medium was used (Fig. 3A). It is hypothesized that plain LC organoids culture media failed to generate MPMOs, whereas GC and CRC possess the necessary components for MPMO cultivation.

To gain a deeper understanding of whether the components in the culture medium play a crucial role in the formation and growth of MPMOs, we cultured MPMOs for 10 days without the aforementioned components and assessed their capacity to grow in their absence. According to our research, the formation of MPMOs will be diminished in number and size if certain components are absent. As with most tumor organoids, the addition of common growth factors such as wnt-3a, noggin, R-spondin-1 to MPMO culture is beneficial to its development. Moreover, it is intriguing to observe that in the absence of FGF10, the number and size of MPMOs decrease significantly, indicating that in addition to adding some common culture factors, FGF10 should also be added to support the growth of MPMOs (Fig. 3B).

Using the above medium scheme, we adjusted the concentration of various factors to create an MPMO medium for MPMOs cultivation. Throughout the cultivation process, we documented the development of each MPMOs by photographing them every 1–2 days (Fig. 3C). Ultimately, we established seven MPM-derived organoids (> 2 passages) from seven patients with an 85.7% success rate. Moreover, one of the samples collected was CPM, and CPMO was produced using the same culture medium (Fig. 2C). After its first passage, the CPMO exhibited significant aging and mortality. We suspect the established culture medium may still lack some crucial factors that support the growth and development of CPMO, but further research is currently unfeasible due to insufficient number of samples.

#### MPMOs recapitulate the histopathological profiles of MPM

**Fig. 4:**
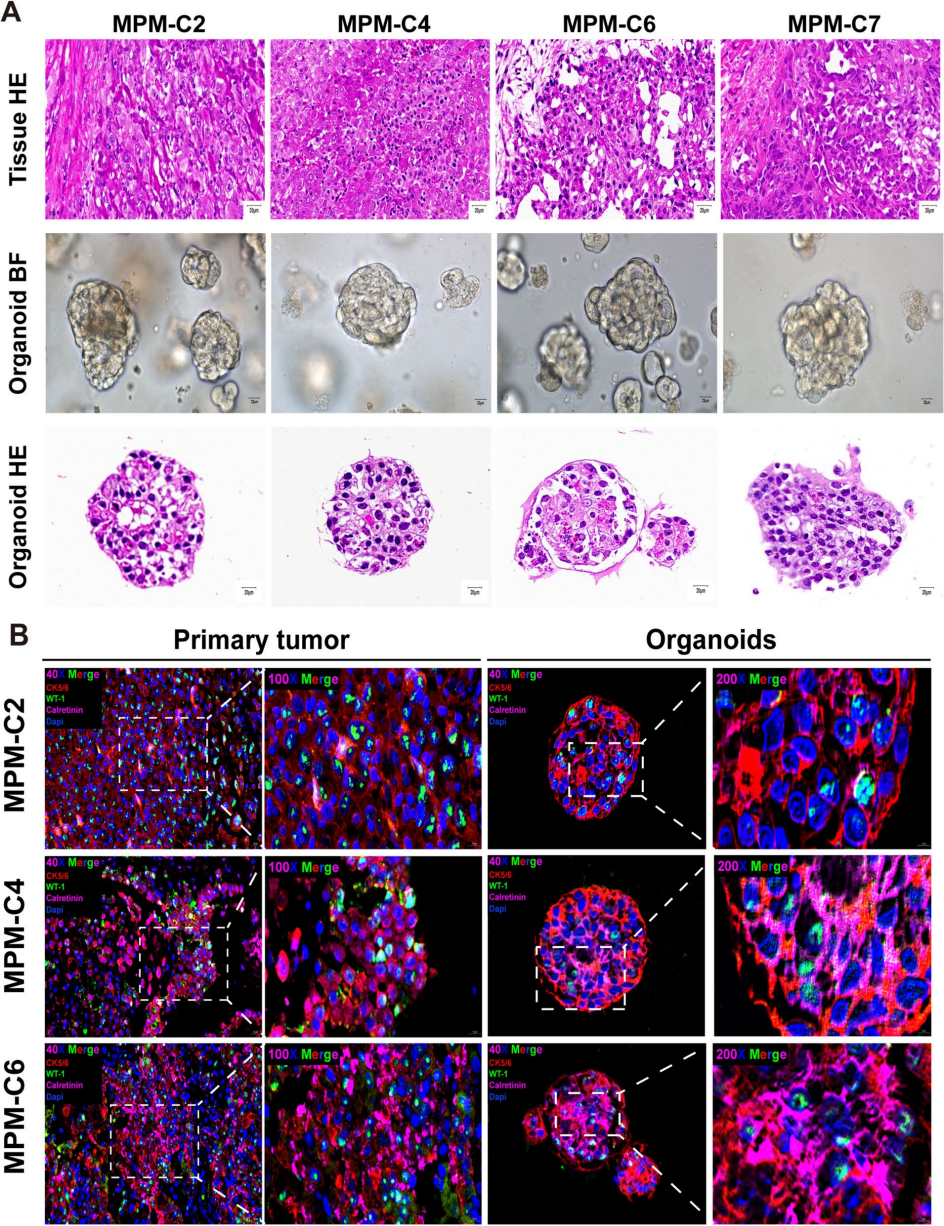
Histological characterization and biomarkers expression analysis of MPMOs. **A** Bright-field (BF) microscopy images of MPMOs together with hematoxylin-eosin (HE) stain histological analysis of MPM tissues and MPMOs. All of the MPMOs were the second generation. HE Scale bar, 20 μm. BF Scale bar, 100 μm. **B** Multiple immuoflurescence staining of cytokeratin 5/6 (CK5/6), Wilms tumor (WT-1), calretinin on MPM-derived organoids and the paimary tumors. CK5/6, red;WT-1, green;Calretinin, pink. 40X Scale bar (20 μm), 100X Scale bar (10 μm), 200X Scale bar (5 μm)

The summary of the characteristics of primitive tumors is the key feature of tumor organoids. To determine whether MPMOs retained the histological patterns of the original tumor samples, paraffin sections were prepared and stained with H&E and immunofluorescence. Light microscopy reveals that MPMO has a three-dimensional structure comprised primarily of various circular-shaped forms. The H&E staining of MPMOs reveals that they are remarkably similar to MPM tissue, containing vacuolar cystic structures in a dense structure (Fig. 4A). The patients in this investigation have been diagnosed with epithelial type malignant mesothelioma. Although their MPMOs have a similar morphology, the morphological characteristics of MPMOs constructed by various patients are distinct. For instance, the structure of MPMOs in cases 2 and 6 is more porous than in cases 4 and 7, with larger and more prominent internal voids (Fig. 3 C, 4A). Moreover, in contrast to the large and small void-like structures of MPMOs, CPMO is predominantly high-density with a large number of small void-like structures (Fig. 2E). Different clinical subtypes of mesothelioma may exhibit substantial differences in the morphology of organoid.

We performed immunofluorescence analyses on four tumor-organoid pairs in order to determine the expression of their diagnostic markers. Currently, CK5/6, WT-1, and calretinin [30, 51, 52] are regarded as potential biomarkers for the clinical and pathological diagnosis of MPM. Among them, CK5/6 resides primarily in the cell membrane, WT-1 resides primarily in the nucleus, and calretinin resides primarily in the cytoplasm. The expression profile of each MPMO marker matched the expression profile of its corresponding primary tumor marker (Fig. 4B, C). In particular, the corresponding MPMO accurately reflects the decreased expression of calretinin in MPM-C2 patients and WT-1 in MPM-C4 patients. Additionally, certain biomarkers are expressed more extensively in MPMOs than in the primary tumor. The preceding data indicate that the differentiation morphology and biomarkers of MPM tissue are conserved in their respective organoids, suggesting that MPMOs reproduce the pathological characteristics of their original tumors.

#### Patient-derived organoid-based xenografts of MPM

To assess the tumorigenicity of MPMOs in vivo, MPMOs were injected subcutaneously into the right axilla of BALB/c nude mice for subcutaneous xenograft experiments and orthotopic xenograft experiments. The success rates of tumor formation were 88.8% (C2 2/3, C6 3/3, C7 3/3 nude mice) and 100% (C2 3/3, C6 3/3, C7 3/3 nude mice), respectively. The subcutaneous xenograft paradigm shows progressive tumor growth.

**Fig. 5:**
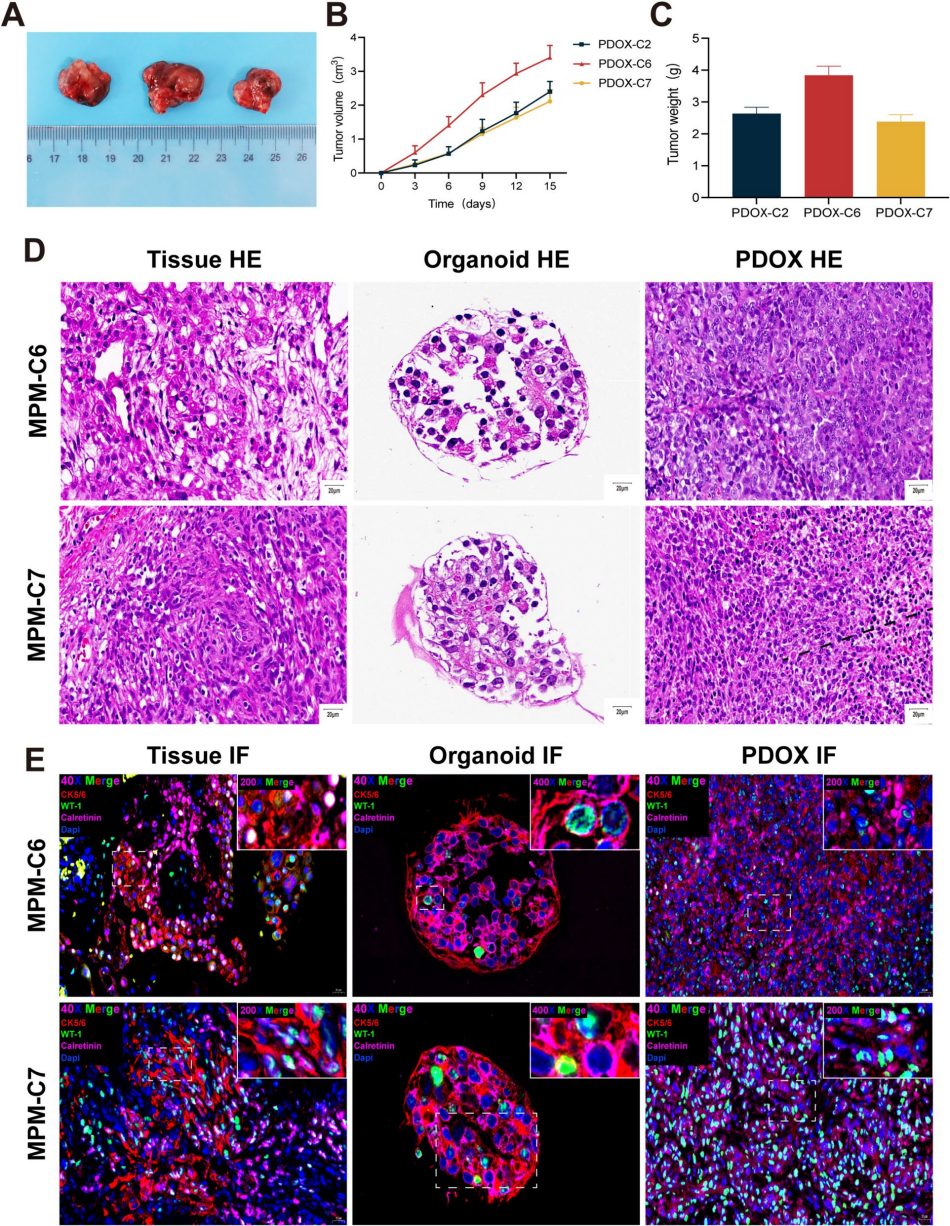
Analysis of MPM organoids xenotransplantation. **A** Representative image of subcutaneous xenograft tumor. **B** The growth curve of subcutaneous xenografts from three different MPM organoids. **C** The weight comparison of subcutaneous xenografts from three different MPM organoids. **D** Histologic assessment of parental tumors, MPM organoids, and xenograft tumors in Hematoxylin-eosin (HE) stain. Scale bar (20 μm). **E** Multiple immuoflurescence staining of cytokeratin 5/6 (CK5/6), Wilms Tumor (WT-1) and calretinin were performed on the parental tumors, MPM organoids, and xenograft tumors. CK5/6, red;WT-1, green;Calretinin, pink. 40X Scale bar (20 μm), 100X Scale bar (10 μm), 200X Scale bar (5 μm), 400X Scale bar (2.5 μm). Data were showed as mean ± SD,Each organoid line was transplanted into three mice

The size of the tumor was measured and recorded every three days until euthanasia and autopsy were performed on day 15 (Fig. 5A, B). C6 grows more rapidly than C2 and C7, whose tumor volume doubling curves and weights are comparable (Fig. 5 C). Similarly, we prepared paraffin sections and performed H&E and immunofluorescent staining to assure that xenografts constructed using MPMOs retained the histological characteristics of their primary tumors. The H&E staining results demonstrated that the tumor tissue of nude mice constructed using MPMOs retained the histological characteristics of the primary tumor, such as large cell volume, diverse morphology, and evident nuclear atypia (Fig. 5D). Furthermore, tumors-organoids-xenografts were subjected to immunofluorescence analysis for determine the expression of their diagnostic markers. The expression profile of each xenograft marker is matched with the expression profiles of the corresponding MPMOs and primary tumor markers (Fig. 5E).

**Fig. 6:**
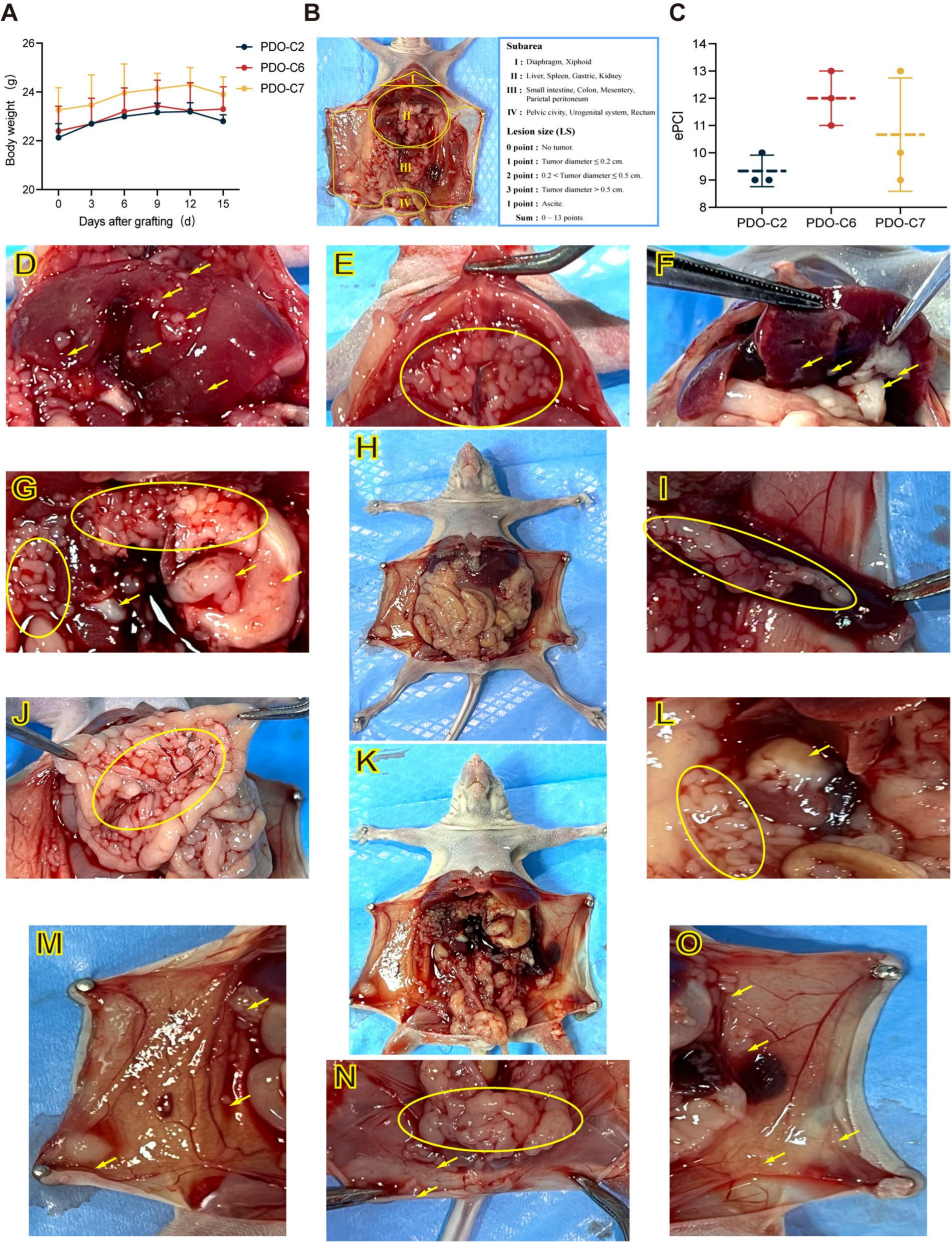
Human derived MPM organoids orthotopic xenotransplantation model (MPM-PDOX). **A** Body weight changes of nude mice after intraperitoneal injection three different MPM organoids. **B** The subarea and scoring of the Experimental Peritoneal Cancer index (ePCI) scoring system [16]. **C** ePCi score of three different PDOX. **D** **- O** The gross pathology of PDOX. (D, F, liver; E, diaphragm; G, pancreas, stomach; H, the images show that the tumor invaded the peritoneal peritoneum and multiple organs of nude mice; I, spleen; J, mesentery; K, retroperitoneal vascular tissue; L, kidney; M,O, peritoneum; N, pelvic cavity.) The images shown are from MPM-C6-O as a representative sample. Data were showed as mean ± SD,Each organoid line was transplanted into three mice

The general condition of nude mice in the intraperitoneal orthotopic xenograft model is normal from the day of transplantation until autopsy. Several nude mice exhibited evident ascites symptoms on the sixth to eighth day of modeling, and certain mice even had bloody ascites. From D1 to D9, the weight of each nude mice increased steadily, and then decreased at D12 (Fig. 6A). On the 15th day after transplantation, we sacrificed the models for autopsy to observe the establishment status, and assessed the tumor formation of MPM using the ePCI score for each orthotopic xenograft model (Fig. 6B, C). In accordance to the gross findings of the postmortem, the tumor has extensive invasion of the abdominal cavity (Fig. 6H), consistent with the biological characteristics of MPM. Based to the ePCI score, we examined numerous regions and organs, including the liver (Fig. 6D, F), diaphragm (Fig. 6E), pancreas (Fig. 6G), stomach (Fig. 6G), spleen (Fig. 6I), mesentery (Fig. 6J), retroperitoneal vascular tissue (Fig. 6K), kidney (Fig. 6L, M, O), and pelvic cavity (Fig. 6 N). Multiple tissues and organs were observed to be invaded by the tumor in the form of a single nodule or an extensive fusion in nude mice. Overall, the PDOX established with MPMOs retains the tumorigenicity and biological behavior characteristics in vivo, and the pathological and histological characteristics of the xenotransplantation are consistent with the original tumor.

#### MPMOs maintain the genetic landscape of originating tumors.

**Fig. 7:**
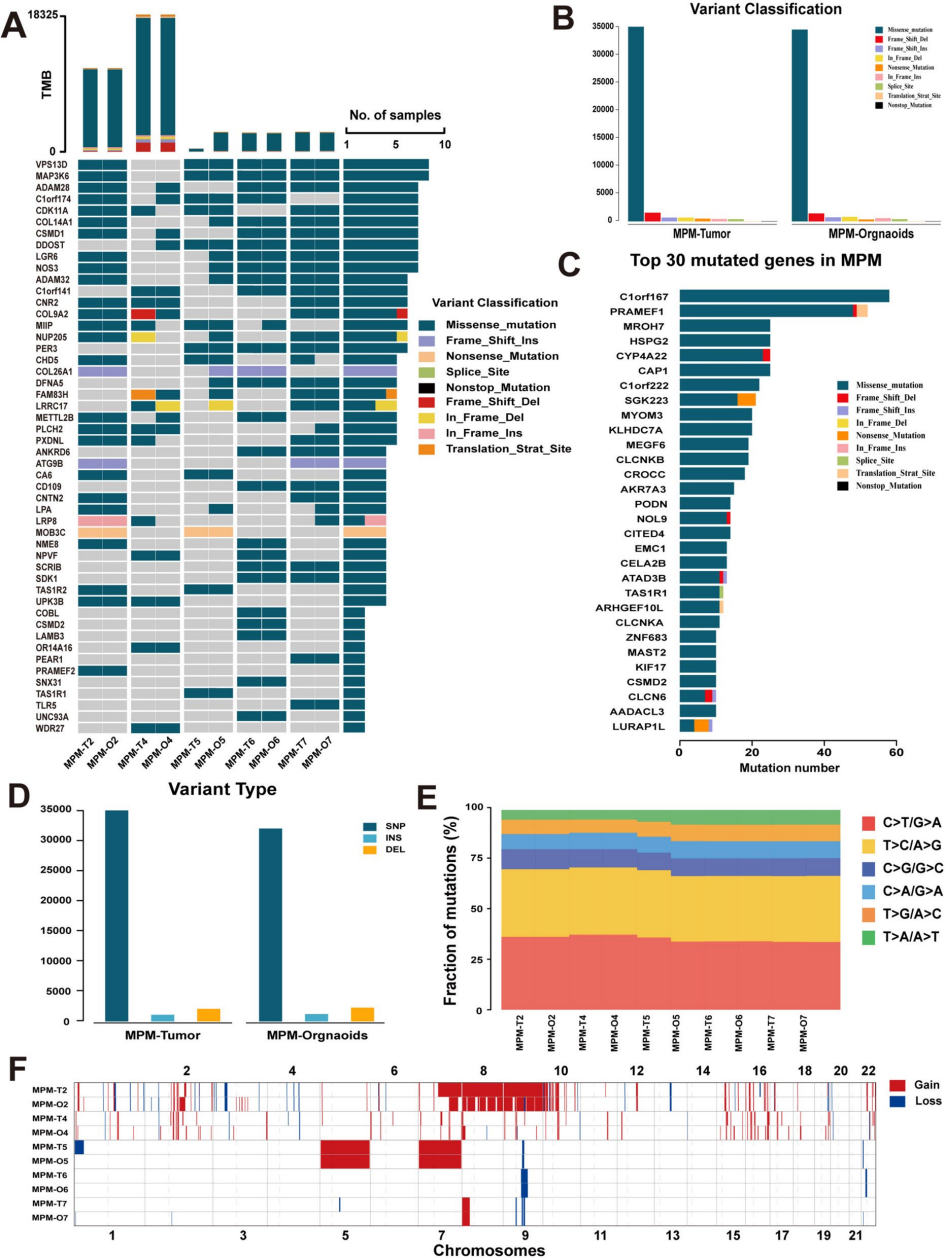
Genetic characterization of malignant peritoneal mesothelioma (MPM) organoids. **A** Somatic genomic landscape of 5 MPMOs (-O) and the corresponding parental tumors (-T). The types of genetic alterations are indicated in the legend. **B** Somatic variation types from MPM tumors and corresponding organoids. **C** The top 30 genes with the highest number of variations in MPM. **D** Proportion of genome mutation types in MPM and corresponding organoids. **E** Histogram illustrating the various contributions of point mutation types in MPMOs(-O) and their respective tumors(-T), the six types of point mutation types are represented. **F** CNVs landscape (red, gain; blue, loss) in MPMOs (-O) and tumor tissues (-T)

We utilized Whole Genome Sequencing (WGS) to determine whether MPMO faithfully replicates the genome map of its tumor origin and the gene mutations found in its parent tumor. On the basis of the sufficient number of organoids obtained for WGS, five pairs of third-generation organoids and their corresponding tissues were selected for sequencing. To quantify the genetic relationship between MPMOs and original tumors, we analyzed their associated missense, splice site, and frameshift mutations, as well as somatic cell base substitution and copy number variations (CNVs). The comparative analysis demonstrates (Fig. 7A) that the tumor mutational burden, (TMB) difference between various MPM patients is substantial; However, the corresponding MPMOs maintain a similar TMB mode, and the majority of somatic mutations in the parent tumor tissue are conserved in the corresponding MPMOs. The most prominent MPM mutations include VPS13D and MAP3K6. They are found in 4/5 pairs of MPM tissues and organoids, and may be essential MPM mutant genes. Through quantitative analysis, a total of 41,267 mutations have been discovered in MPM tumor tissue, including 83.6% missense mutations, 6.7% frameshift mutations, 1.9% deletion mutations, 1.3% nonsense mutations, and other mutation types. Similar outcomes were observed in the corresponding organoids (Fig. 7B). In addition, we exhibited the top 30 genes with the highest mutation number for MPM (Fig. 7C).

Variation in the structural composition of the genome is of considerable importance in the study of illness occurrence and progression. Therefore, we analyzed the predominant mutation type in the MPM genome and MPMOs, which was SNP (Fig. 7D). As the most common mutation, SNP primarily refers to the variation of the genome caused by a single nucleotide, which can lead to in DNA sequence polymorphism between individuals. In order to determine whether MPMOs retain the genomic characteristics of their progenitor tumors, we compare the point mutation types. Quantified point mutation reveals that the most prevalent point mutations in MPM are G > A/C > T and T > C/A > G over conversion, while T > A/A > T is the least common, while the distribution of MPMOs point mutation retains the majority of its features (Fig. 7E). In addition, CNV analysis was conducted to determine whether MPMO retained the genomic features of the primary tumor. It shows that the paired MPMO retained the patterns of chromosomal gain and loss observed in the primary tumor, as well as exhibiting comparable DNA CNV patterns (Fig. 7F). According to the findings of WGS, MPMOs can accurately reflect the genomic characteristics from different patients’ tumors. With the aid of MPMOs, it is possible to investigate the genetic characteristics of the occurrence and progression of MPM in greater detail.

#### MPM patient-derived 2D cell lines

**Fig. 8:**
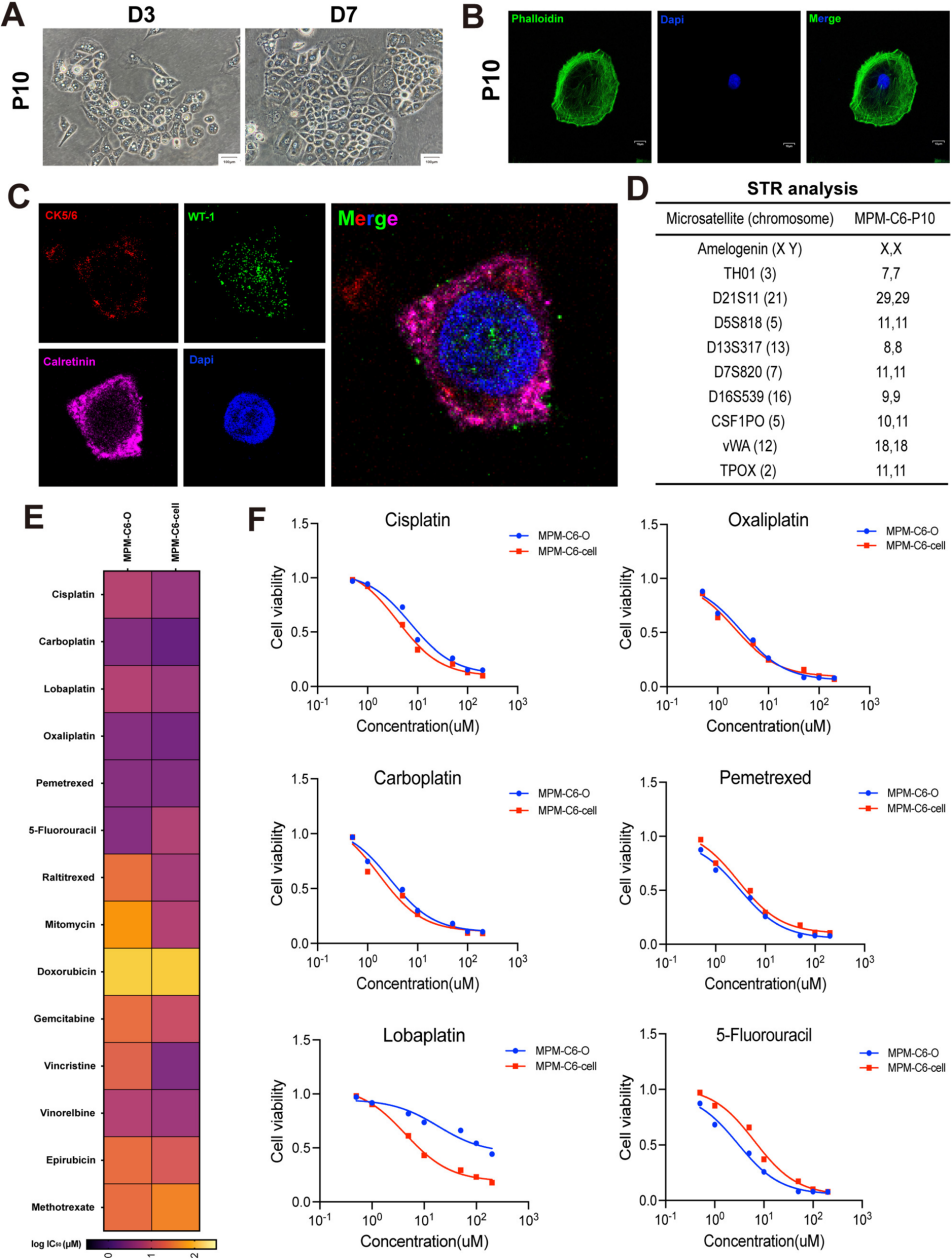
Cytological characteristics of malignant peritoneal mesothelioma (MPM) primary cell lines. **A** Morphology was showed under the inverted phase contrast microscope of MPM-C6 primary cell. Scale bar, 100 μm. **B** The MPM-C6 primary cell morphology was revealed by phalloidin staining. Scale bar, 10 μm. **C** Multiple immuoflurescence staining of cytokeratin 5/6 (CK5/6), Wilms Tumor (WT-1), calretinin on MPM-C6 primary cells. CK5/6, red;WT-1, green;Calretinin, pink. Scale bar, 10 μm. **D** The genomic authentication of the MPM-C6 primary cell was confirmed by the short tandem repeats (STRs). **E** Heat map of logIC50 values for 14 anticancer drugs used to treat MPM-C6 primary cell and related MPM organoids. Putative targets of the tested anticancer drugs are listed on the left. **F** Representative dose response curves of the MPM-C6 primary cell and related MPM were treated with first-line chemotherapy drugs alone. Data were showed as mean ± SD and each experiment were performed in triplicate

During the process of establishing MPMO, we also attempted to establish the primary cell line of MPM by cultivating the primary cells of each patient individually. Initially, the primary MPM cells of each case were predominantly monolayer adherent cells that were grown and passaged as 2D cell lines. Unfortunately, the majority of 2D primary cell lines from patients ceased growing and died after the third generation. Ultimately, only the Case 6 primary cell line can be cultured for more than 10 generations. The MPM primary cells proliferate in a monolayer adhesion phase, with an average passage every 5–7 days. Inverted phase contrast microscopy reveals that the cell morphology is polygonal and varies in size, with multinucleated and macro-nucleated nuclei visible (Fig. 8A). The phalloidin staining revealed the morphological diversity of MPM cells, such as the irregular structure on the cell membrane (Fig. 8B). We examined the expression of pertinent biomarkers via immunofluorescence staining to confirm that the primary cell line is composed of MPM cells. The cell was discovered to have traits resembling those of the original tumor tissue and to express calretinin, WT-1, and cytokeratin 5/6 (Fig. 8C). In addition, the most common cause for the failure of primary cell culture is contamination and replacement by immortal cell lines. Therefore, it is clarified further that this situation does not occur in cultured MPM cells, so we performed STR genotyping analysis. The results of STR indicate that the MPM primary cell line we created does not match any of the known immortal cell lines (Fig. 8D, Supplementary Fig. 1).

Previous research has demonstrated that primary cells derived from patients have an amount of predictive ability in assessing drug efficacy. In order to evaluate the viability of MPMOs and single cell chemotherapy drug sensitivity tests, it was necessary to execute drug screenings with established primary cells and their associated MPMOs. For drug selection, we primarily evaluate the drugs included in the present first-line and second-line treatments for MPM, with pemetrexed in combination with platinum-based chemotherapy drugs constituting the primary clinical treatment therapy for MPM (Supplementary Table 4). The results of the drug screenings revealed that the commonly used chemotherapy drugs pemetrexed and the majority of platinum in first-line treatment have significant inhibitory effects on MPMO and primary cells derived from MPM-C6 patients, with carboplatin having the lowest half-maximal inhibitory concentrations (IC50) value (Fig. 8E, F). According to the results, the IC50 of MPM primary cells is generally lower than that of MPMO, indicating that they are more sensitive to drug effects. This may be due to the fact that the growth of primary cells consists primarily of monolayer planar growth, which interacts better with drugs, resulting in increased drug sensitivity (Fig. 8E). Moreover, it is interesting to observe that the majority of platinum compounds have a significant inhibitory effect on MPMO-C6, whereas lobaplatin was unable to satisfy the IC50 requirement at the maximum concentration we set for the drug. In contrast, the IC50 of the drug remained low in primary cells (Fig. 8F). Consequently, we suggest that using early cultured MPMOs for drug screenings may produce results that are more representative of the actual efficacy of patients than using primary cells.

#### Drug sensitivity test on MPM-derived organoids

**Fig. 9:**
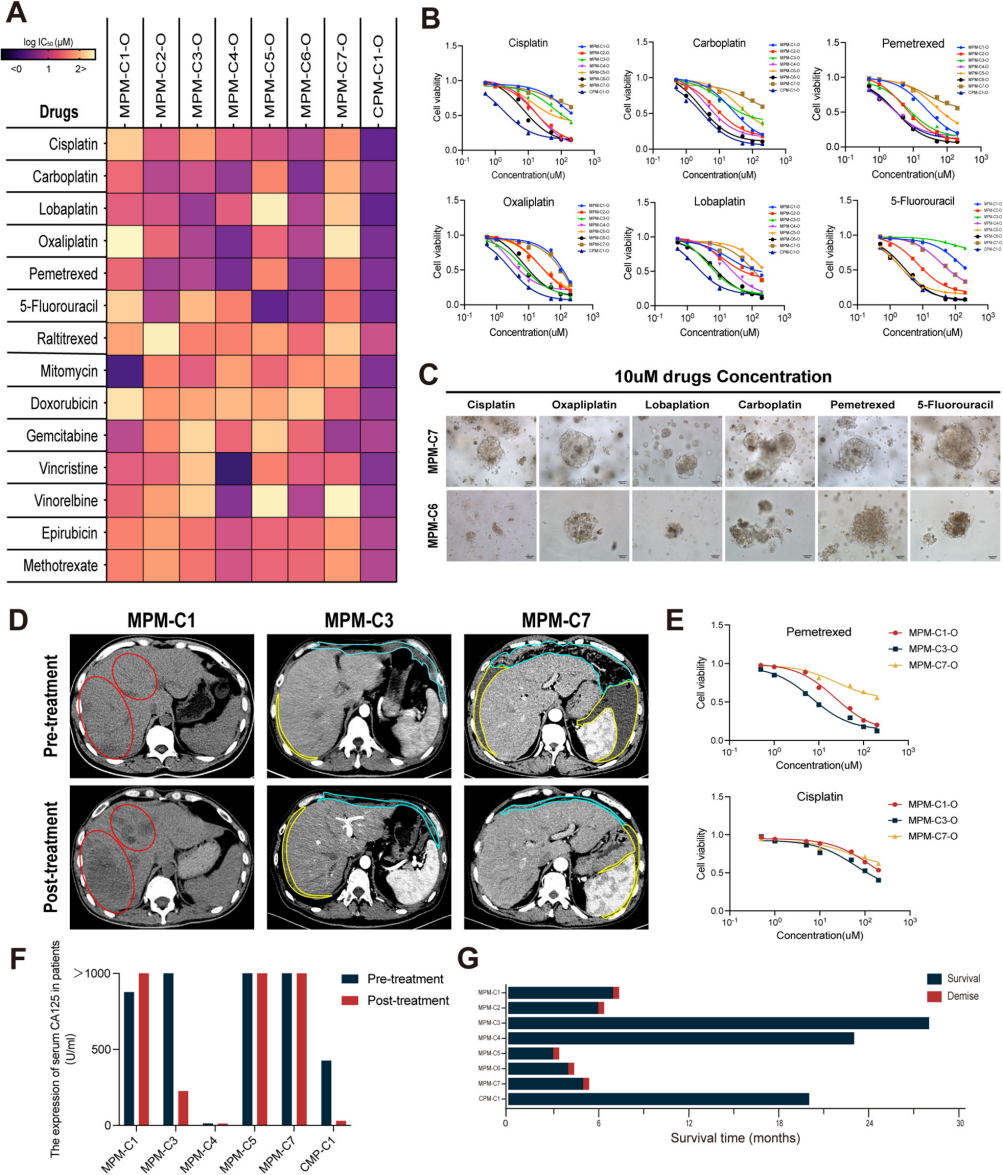
Drug response in malignant peritoneal mesothelioma (MPM) organoid lines. **A** Heat map of logIC_50_ values of 14 anticancer drugs used to treat seven MPMOs and a CPMO from 8 patients. All organoids are of the first generation. **B** Dose response curves of first-line chemotherapy drugs for MPMOs and CPMO. **C** MPM organoids morphology after first-line chemotherapy drugs treated were showed under the inverted phase contrast microscope. The image shown are from MPM-C7-O (drug resistance) and MPM-C6-O (drug sensitivity) as a representative sample. **D** CT scan images show the tumor status of MPM patients during treatment. Primary tumor (circled in cyan), metastatic lesion (circled in red), ascites (circled in yellow). **E** Fitted dose–response curves illustrating the distinct responses of MPMOs to pemetrexed and cisplatin. **F** The serum CA125 levels in different MPM patients pre and post treatment. **G** The overall survival of the MPM and CPM patients in this study. Data were showed as mean ± SD and each experiment were performed in triplicate

Currently, MPM can only be treated with the same drug regimen as malignant pleural mesothelioma, but drug resistance and individual differences have become impediments to MPM treatment. Multiple studies have considered patient-derived organoids as a preclinical model that may represent the actual efficacy of patients, and this model is expected to replace patients in drug screening, thereby enabling the development of personalized medication regimens for patients. In order to assess the efficacy of preclinical models for anticancer drug response, we selected 14 anticancer drugs currently used for the treatment of MPM and conducted drug screening on the established MPMOs (Supplementary Tables 4, Fig. 9A). From the results, it shown that MPM-C1-O, MPM-C5-O and MPM-C7-O have a high IC50 against pemetrexed and platinum medicines, and the drug has a poor inhibitory effect on the proliferation of MPMOs. But MPM-C3-O, MPM-C4-O and MPM-C6-O are significantly inhibited by platinum pharmaceuticals and pemetrexed (Fig. 9B). Under the microscope, MPM-C6-O demonstrates significant organoid mortality or growth inhibition in response to drug treatment. Due to the insensitivity of MPM-C7-O to the majority of the drugs tested in this study, a circumstance similar to that of MPM-C6-O was not observed, and the organoids of MPM-C7 continued to grow under the influence of the drugs (Fig. 9C). Although pemetrexed and platinum drugs are the preferred drugs for MPM treatment, our results demonstrate that different patients have different sensitivities to different platinum drugs (Fig. 9B), and even some patients’ MPMOs have more significant inhibitory effects on some second-line drugs, including MPM-C1 for mitomycin, MPM-C4 for vincristine, and MPM-C5 for 5-fluorouracil (Fig. 9A). Moreover, it is intriguing to observe that the CPMO we have identified has a significant inhibitory effect on organoid growth when administered in low doses. We hypothesize that this result may be due to the fact that CPM is a low-grade malignant tumor with a favorable prognosis, resulting in a high sensitivity to chemotherapy medications. Unfortunately, we were unable to gather more CPM instances for additional verification.

Next, we assessed the drug sensitivity results of MPMOs and the actual treatment status of MPM patients. For retrospective analysis, we collected post-treatment prognostic information for each patient in this study, including CT scan image (Fig. 9D), serum CA125 levels (Fig. 9F), and overall survival time (Fig. 9G). In this study, all patients were administered the pemetrexed + cisplatin regimen, but its efficacy differed significantly between patients. During the treatment period, metastatic lesions on the liver of MPM-C1 patient continued to increase progressively (circled in red), and the serum CA125 level continued to climb. Even though MPM-C7 patient received CRS + hiPeC treatment, serum CA125 levels increased and tumor recurrence occurred (circled in cyan) during the treatment period. In particular, MPM-C3 patient exhibited no significant changes in the primary tumor lesion (circled in cyan) or ascites (circled in yellow) on CT scan pre- and post-treatment, but the serum CA125 level was decreased significantly. Combined analysis with overall survival rate, patient MPM-C1 and MPM-C7 are exhibited significant resistance to the pemetrexed + cisplatin regimen, and both patients continued to progress with MPM during treatment. For MPM-C3 patient, although no reduction in lesion was observed on CT scans, when analyzed in conjunction with CA125 and overall survival time, who is still sensitive to the pemetrexed + cisplatin regimen treatment. However, for MPM-C4 and CPM-C1 patients receiving CRS + hiPeC treatment, the lack of evaluable lesions and serological markers, the absence of evidence of recurrence during the follow-up period, and survival time demonstrate that both patients are sensitive to treatment. Overall, the actual efficacy of treatment in the patients in this study was consistent with the drug sensitivity results of MPMOs and CPMO (Fig. 9A, B and E). Unfortunately, we were unable to analyze all of the patients in this study, mainly due to the fact that MPM-C5 and - C6 patients were in a terminal stage when MPM was discovered, and their condition progressed rapidly, so we were unable to evaluate their treatment.

## Discussion

Malignant peritoneal mesothelioma is a disease with a wide spectrum of clinical manifestations. It originates in the peritoneal membrane and is typically distributed throughout the abdominal cavity [53, 54]. Currently, the most common treatment for MPM is cell reduction surgery (CRS) combined with intraperitoneal hyperthermia chemotherapy (hiPeC), with the majority of medications adapted from the regimen for malignant pleural mesothelioma [6, 17–19]. However, there are substantial distinctions between MPM and malignant pleural mesothelioma, which may impact the efficacy of treatment [54]. Whereas in fact, experimental treatment for patients with recurrent, drug-resistant, or late-stage inability to undergo CRS can only be administered by clinical doctors, resulting in an exceedingly poor prognosis. In addition, a large number of MPM studies have been hampered by the lack of appropriate research models, resulting in the majority of medical research on MPM focusing on case reports and retrospective analyses of efficacy, ultimately leading to a large number of unanswered questions regarding MPM, such as its primary pathogenic factors, primary driving pathways, drug targets, and treatment effects [6, 7, 17, 55, 56].

PDO has garnered significant attention as a novel generation of in vitro tumor models. Previous studies have shown a number of optimal applications of organoid culture to personalized cancer medications [34–42]. It can not only be used to predict the clinical results of anticancer drugs in individual patients, but also possesses the qualities of stable cultivation, flexible operation, faithful restatement of patient-specific characteristics and genetic landscape of primary tumors, notably tumor heterogeneity, and overcoming the limitations of existing models [57–59]. In summation, PDO is a novel tumor model with limitless potential in many categories, including future disease research and precision medicine.

In this study, we described a 3D culture system for generating MPM organoids in vitro from patient samples. Through histopathological examination of MPMOs and primary tumor, it is confirmed that our MPMOs retains the primary tumor’s histopathological characteristics. On this basis, we developed MPM-PDOX by MPMOs, which replicates the pathological development of clinical patients in a substantial manner. According to the orthotopic transplantation model of MPMOs, the tumor infiltrates multiple organs and the peritoneum, producing multiple nodular tumors. Some PDOXs also exhibit bloody ascites, restoring in full the high malignant potential and aggressive invasion and metastasis behavior of MPM. Moreover, compared to previous PDX models, the MPM-PDOX constructed with MPMOs has a high success rate and is greatly usable. Future research on the pathological mechanisms and clinical interventions of MPM will be facilitated by the combination of MPMOS and PDOX models. 

Precision medicine is the foundational cancer strategy for the past and future. Cancer patients should immediately utilize medications that are sensitive, specific, and effective for each patient. However, for tumor types such as MPM that have only a few known drug targets or genomic characteristics, it is still challenging to find treatment options that are appropriate for different individuals. Consequently, the current treatment for MPM can only be derived from the treatment strategy for malignant pleural mesothelioma. A study on the combination of pemetrexed and platinum chemotherapy in the treatment of MPM showed that for patients with unresectable peritoneal malignant mesothelioma, the disease control rate can reach 71%, but the median survival time is only 13.1 months [60]. Another clinical trial involving pemetrexed and gemcitabine was conducted. The median patient survival time rose to 26.8 months, but the overall effectiveness rate was only 15%. The remaining 60% of patients experience serious adverse effects [49]. It is apparent that there is very little understanding of MPM, and that only experiential treatment can be expected to benefit MPM patients. MPM-PDO cultivation opens up possibilities for future MPM to satisfy this demand, as the system faithfully replicates the original tumor and can be used to replace patients for drug testing [37, 59, 61]. In our study, drug screening of MPMOs revealed that drugs with the same mechanism of action exhibited inconsistent sensitivity to the same MPMOs or the same drug exhibited inconsistent sensitivity to various MPMOs, indicating that patients exhibited drug-specific sensitivity. We conducted a retrospective analysis through tracking each patient’s prognosis and comparing it to the drug sensitivity of the corresponding MPMO. We observed that MPM-C1 and MPM-C7 patients continued to experience tumor growth during treatment, with an ultimate survival time of approximately 6 months. However, for MPM-C3 and MPM-C4 patients, this treatment regimen demonstrated great sensitivity, and the survival period exceeded 20 months. The efficacy results for these patients are consistent with the results exhibited by MPMOs through drug screening. This demonstrates that the combination of the chemotherapy medications pemetrexed and platinum cannot benefit every patient. In the future, the use of MPMOs as a substitute for patients for in vitro efficacy analysis will provide the opportunity to further personalize treatment regimens for patients and increase their survival.

In conclusion, we are one of the pioneering organizations that established the MPM-PDO platform. In the study, we demonstrated the prospective use of MPMOs as a promising new preclinical model representative of specific patients. In terms of histopathology, genome characteristics, mutation characteristics, and intratumoral heterogeneity, the extensive characteristics of MPMOs indicate that they maintain the landscape of their progenitor tumor. However, there are still limitations in this discipline that require attention at this stage. First, the limited sample size is the most significant limitation, and more clinical validation cases are required to evaluate the efficacy and safety. Second, during the long-term organoids culture process, heterogeneous tumor cells may progressively evolve or disappear, thereby affecting the precision. Therefore, we suggested using early PDO to identify efficacious drugs. Thirdly, the MPM patients in this study have just a single subtype, and it is anticipated that in the future, more cases of various subtypes will be required to optimize and enhance the cultivation program.

This study provides an example of establishing a personalized treatment strategy guided by MPMOs. In the future, we will further investigate and optimize the cultivation of MPMOs, increase the number of samples, further optimize the process, make MPMOs more popular, improve the success rate of cultivation, and verify the feasibility of the cultivation plan in various pathological subtypes, thereby significantly expanding the preclinical research toolbox on MPM.

### Supplementary Information

**Additional file 1: Supplementary Table 1.** The clinical information of malignant and cystic peritoneal mesothelioma patients. **Supplementary Table 2.** Recipe of the malignant peritoneal mesothelioma organoid culture media. **Supplementary Table 3.** The antibody information. **Supplementary Table 4.** The chemotherapy drugs information/ **Supplementary Figure 1. **The STR analysis of MPM primary cell line from MPM-C6.

## Data Availability

The data that support the findings of this study are available from the corresponding author upon reasonable request.

## Abbreviations

MPM: Malignant peritoneal mesothelioma
MPMOs: Malignant peritoneal mesothelioma organoids
PDO: Patient-derived organoids
PDOX: Patient-derived organoids xenograft
IF: Immunofluorescence
ePCI: Experimental peritoneal cancer index
H&E: Hematoxylin and eosin
