# Supplementary material for: Organoids derived from patients provide a new opportunity for research and individualized treatment of malignant peritoneal mesothelioma
Source: Mol Cancer. 2024 Jan 10;23:12. doi: 10.1186/s12943-023-01901-z (PMC10782772; doi:10.1186/s12943-023-01901-z)

**Supplementary Information**

**Supplementary Table 1.** The clinical information of malignant and cystic peritoneal mesothelioma patients

| NO. of Patient | Gender | Age | Histotype | CA125  (U/ml) | Smoking Score | Asbestos Exposure | PCI*** |
| --- | --- | --- | --- | --- | --- | --- | --- |
| MPM-C1 | Male | 48 | MPM* | 877.40 | None | None | 31 |
| MPM-C2 | Male | 58 | MPM* | 40.61 | 800 | None | 33 |
| MPM-C3 | Male | 45 | MPM* | 2021.00 | None | None | 37 |
| MPM-C4 | Male | 65 | MPM* | 13.21 | 600 | None | 24 |
| MPM-C5 | Female | 55 | MPM* | 7602.00 | None | None | 31 |
| MPM-C6 | Male | 62 | MPM* | 62.05 | 150 | None | 32 |
| MPM-C7 | Male | 65 | MPM* | 2219.00 | 800 | None | 36 |
| CPM-C1 | Male | 52 | CPM** | 426.7 | None | None | 39 |

* MPM: malignant peritoneal mesothelioma; **CPM: cystic peritoneal mesothelioma; ***PCI : peritoneal carcinomatosis index

**Supplementary Table 2.** Recipe of the malignant peritoneal mesothelioma organoid culture media

| Regents | Source | Catalog No. | Concentration |
| --- | --- | --- | --- |
| Advanced DMEM/F12 | Thermo Fisher Scientific | 11320-033 | - |
| Penicillin-Streptomycin | Gibco | 15070-063 | 1% |
| Primocin® | InvivoGen | ant-pm-05 | 1% |
| HEPES | Gibco | 15630-080 | 10mM |
| GlutaMAX™ Supplement | Gibco | 35050-061 | 10mM |
| B-27™ Supplement | Invitrogen | 17504044 | 1X |
| N2 Supplement | Invitrogen | 17502048 | 1X |
| Nicotinamide | Sigma | N0636 | 20mM |
| N-Acetyl-L-cysteine | Sigma | A9165 | 1mM |
| EGF | MedChemExpress | HY-P7109 | 50ng/ml |
| Prostaglandin E2 | Sigma | P0409 | 10nM |
| Gastrin I | MedChemExpress | HY-P1097 | 1nM |
| Transferrin human | Sigma | T1147 | 10ug/ml |
| Insulin | MedChemExpress | HY-P0035 | 7.5ug/ml |
| Wnt-3a | R&D Systems | 5036-WN-010 | 100ng/mL |
| Noggin | Abcam | ab73756 | 100ng/mL |
| R-Spondin1 | MedChemExpress | HY-P7114 | 1ug/mL |
| FGF10 | Peprotech | AF- 100-26 | 200ng/mL |
| A83-01 | MedChemExpress | HY-10432 | 2uM |
| SB202190 | MedChemExpress | HY-10295 | 10uM |
| Y27632 | MedChemExpress | HY-10071 | 10uM |
| CHIR999021 | MedChemExpress | HY-10182 | 2.5uM |
| BME, Type2 | R&D Systems | 3536-005-02 | - |

**Supplementary Table 3.** The antibody information

| Target | Source | Catalog No. | Dilution |
| --- | --- | --- | --- |
| Cytokeratin 5/6 | Abcam | ab64081 | 1:100 |
| WT-1 | Cell Signaling Technology | #83535 | 1:100 |
| Calretinin | Cell Signaling Technology | #92635 | 1:200 |
| FITC Goat Anti-Rabbit IgG | ABclonal Technology | AS011 | 1:300 |
| Cy3 Goat Anti-Rabbit IgG | ABclonal Technology | AS007 | 1:300 |
| 647-conjugated Goat  Anti-Rabbit IgG | ABclonal Technology | AS060 | 1:300 |

**Supplementary Table 4.** The chemotherapy drugs information

| Chemicals | Source | Catalog No. | Drugs Target |
| --- | --- | --- | --- |
| Cisplatin | MedChemExpress | HY-17394 | DNA synthesis |
| Carboplatin | MedChemExpress | HY-17393 | DNA synthesis |
| Lobaplatin | MedChemExpress | HY-105930 | DNA synthesis |
| Oxaliplatin | MedChemExpress | HY-17371 | DNA synthesis |
| Pemetrexed | MedChemExpress | HY-10820 | Dihydrofolate reductase, Thymidylate synthase, glycinamide riboneucleotide transformylase |
| 5-Fluorouracil | MedChemExpress | HY-90006 | Thymidylate synthase |
| Raltitrexed | MedChemExpress | HY-10821 | Thymidylate synthase |
| Mitomycin | MedChemExpress | HY-13316 | DNA synthesis, polymerization |
| Doxorubicin | MedChemExpress | HY-15142A | DNA topoisomerase Il |
| Gemcitabine | MedChemExpress | HY-17026 | DNA polymerase |
| Vincristine | MedChemExpress | HY-N0488A | Microtubule |
| Vinorelbine | MedChemExpress | HY-12053AS | Microtubule |
| Epirubicin | MedChemExpress | HY-13624 | DNA/RNA synthesis, Topoisomerase |
| Methotrexate | MedChemExpress | HY-14519 | Dihydrofolate reductase |

**Supplementary Figure 1.** The STR analysis of MPM primary cell line from MPM-C6


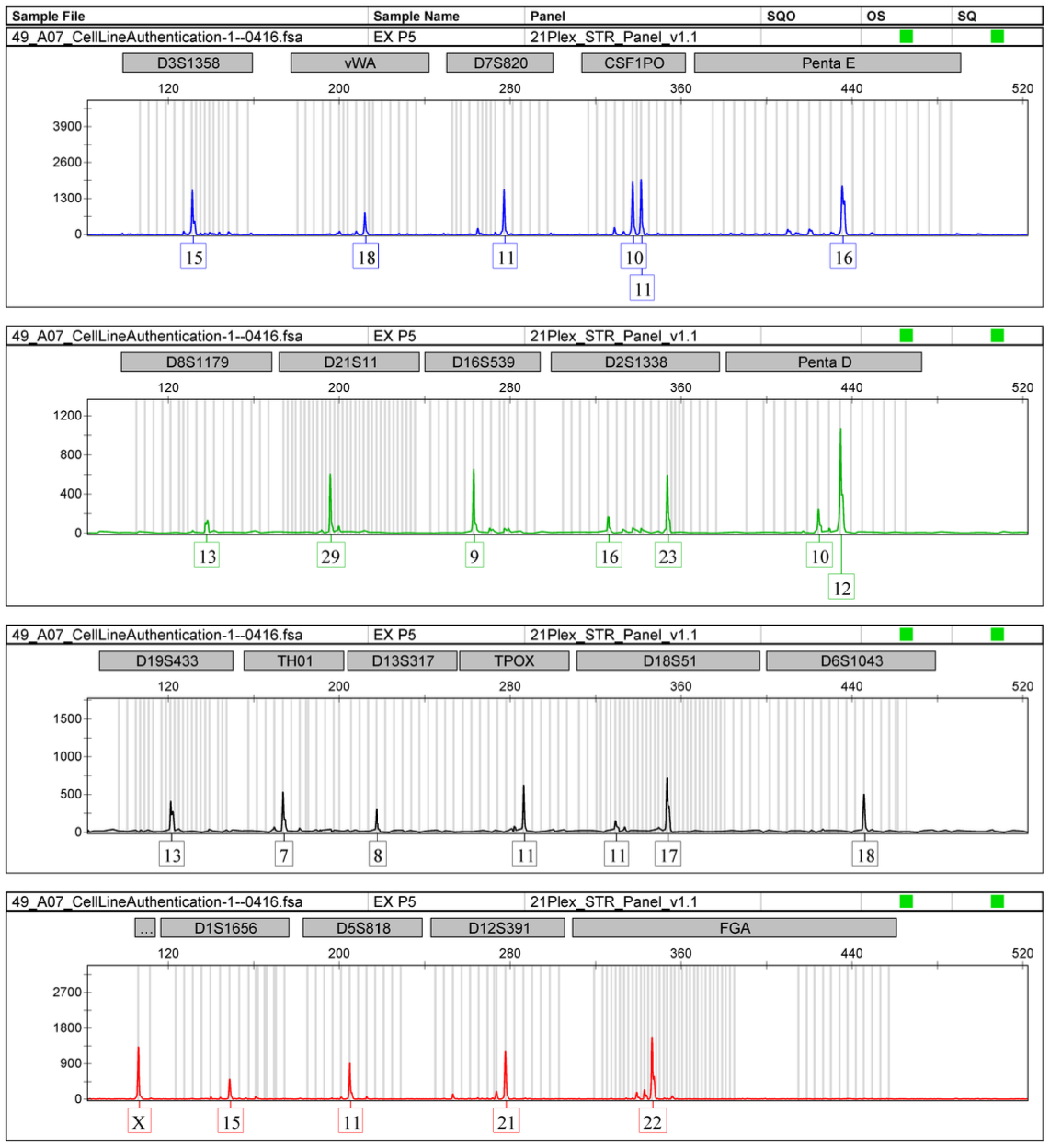

Supplement: Supplementary file 1 — Additional file 1: Supplementary Table 1. The clinical information of malignant and cystic peritoneal mesothelioma patients. Supplementary Table 2. Recipe of the malignant peritoneal mesothelioma organoid culture media. Supplementary Table 3. The antibody information. Supplementary Table 4. The chemotherapy drugs information/ Supplementary Figure 1. The STR analysis of MPM primary cell line from MPM-C6. [file 12943_2023_1901_MOESM1_ESM.docx]
